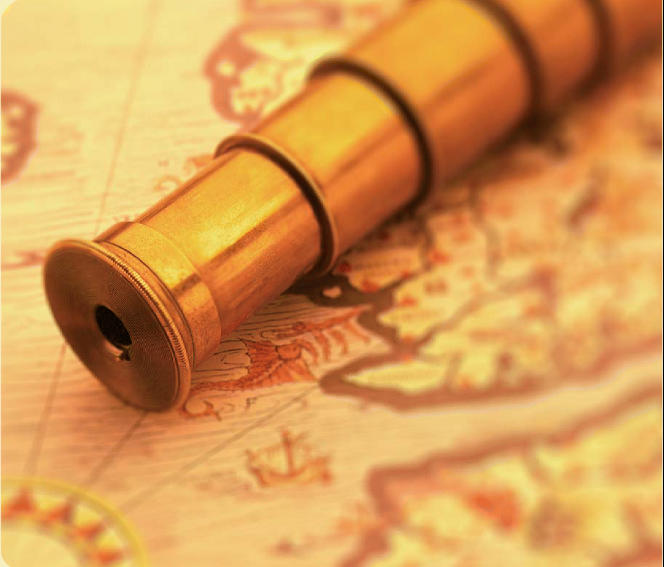# NIEHS DISCOVER Centers

**Published:** 2006-05

In January 2006, the NIEHS announced its newest Centers program: the Disease Investigation through Specialized Clinically Oriented Ventures in Environmental Research (DISCOVER) Centers. The fundamental intent of these new centers is to speed the translation of mechanistically driven research into clinical application and improved public health through a combined interdisciplinary effort in basic science and patient-oriented research. These centers will address long-standing critical disease-relevant issues by applying environmental health sciences to understand human disease and dysfunction.

A DISCOVER Center must reflect an integrated research enterprise that will advance understanding of how environmental stimuli interact with biologic processes to either preserve health or cause disease. Centers will bring together an interdisciplinary research team focused on a defined human disease or dysfunction in which there is adequate justification for the role of environmental stressors in influencing disease etiology, progression, prognosis, or population distribution. The research conducted by a DISCOVER Center will capitalize on multiple aspects of environmental health sciences research including exposure biology, environmental genetics and genomics, patient-oriented clinical research and public health sciences, such as epidemiology, as well as computational and engineering approaches to define the functional contributions of environmental and genetic determinants in

- assessing the risk of developing disease
- identifying the underlying physiologic mechanisms in disease pathogenesis and progression
- characterizing disease phenotype
- understanding the environmental and endogenous factors that affect the distribution of disease in populations
- applying the knowledge gained to develop therapeutic, diagnostic, prognostic, and preventative strategies.

The NIEHS intends to commit a total of $9 million dollars for competitive awards made in fiscal years 2007 and 2008. We anticipate funding four to six new DISCOVER Center grants.

## Contacts

**The DISCOVER Project Team,** discover@niehs.nih.gov

**Dr. David Balshaw** | balshaw@niehs.nih.gov

**Dr. Kimberly Gray** | gray6@niehs.nih.gov

**Dr. Jerry Heindel** | heindelJ@niehs.nih.gov

**Dr. Claudia Thompson** | thomps14@niehs.nih.gov

## Figures and Tables

**Figure:**